# Report of Deaths in Buffalo for the Month of April, 1855

**Published:** 1855-06

**Authors:** J. Root

**Affiliations:** Health Physician


### ART. VII.—*Report of Deaths in Buffalo for the month*

| DISEASES. | Males. | Females. | Total. | AGE. 1 year and Under. Male. | Female. | 1 to 2 years. Male. | Female. |
|---|---|---|---|---|---|---|---|
| Accident, | 1 | .... | 1 | .. | .. | .. | .. |
| Atrophia, | 1 | 1 | 2 | .. | 1 | .. | .. |
| Apoplexy, | 1 | .... | 1 | .. | .. | .. | .. |
| Asthma, | 1 | .... | 1 | .. | .. | .. | .. |
| Consumption, | 16 | 8 | 24 | .. | .. | 1 | .. |
| Convulsions, | 8 | 3 | 1 | 4 | 2 | 2 | .. |
| Croup, | 2 | 1 | 3 | .. | .. | 1 | .. |
| Cyanocis, | 1 | .... | 1 | .. | .. | 1 | .. |
| Cholera Morbus, | .... | 1 | 1 | .. | .. | .. | .. |
| Chronic Pleurisy, | 1 | .... | 1 | .. | .. | .. | .. |
| Congestion of Brain, | 1 | .... | 1 | .. | .. | .. | .. |
| " " Lungs, | 1 | 1 | 2 | .. | .. | .. | .. |
| Canker, | .... | 1 | 1 | .. | .. | .. | .. |
| Drowned, | 2 | .... | 2 | .. | .. | .. | .. |
| Dropsy, | 1 | 2 | 3 | .. | .. | .. | .. |
| " of Brain, | 1 | .... | 1 | 1 | .. | .. | .. |
| Debility, | 3 | 5 | 8 | 2 | 3 | .. | .. |
| Dentition, | 1 | 1 | 2 | .. | 1 | 1 | .. |
| Dysentery, | 1 | 1 | 2 | .. | .. | 1 | .. |
| Diarrhœa, | .... | 1 | 1 | .. | 1 | .. | .. |
| Erysipelas, | 2 | 1 | 3 | 1 | .. | .. | .. |
| Fever, Typhoid, | 1 | .... | 1 | .. | .. | .. | .. |
| " Typhus, | .... | 1 | 1 | .. | .. | .. | .. |
| " Scarlet, | 2 | 1 | 3 | .. | .. | 1 | .. |
| " Congestive, | .... | 1 | 1 | .. | .. | .. | .. |
| Gastritis, | 1 | .... | 1 | .. | .. | .. | .. |
| Hydrocephalus, | 1 | 1 | 2 | .. | .. | 1 | 1 |
| Hæmorrhagia Uteri, | .... | 1 | 1 | .. | .. | .. | .. |
| Inflammation of Bowels, | 1 | .... | 1 | .. | .. | 1 | .. |
| Inanition, | .... | 1 | 1 | .. | .. | .. | .. |
| Laryngismus Stridulus, | .... | 1 | 1 | .. | .. | .. | 1 |
| Marasmus, | 4 | 2 | 6 | 2 | 1 | .. | .. |
| Nervous Prostration, | 1 | .... | 1 | .. | .. | .. | .. |
| Old Age, | .... | 4 | 4 | .. | .. | .. | .. |
| Pneumonia, | 5 | 3 | 8 | 2 | 1 | .. | 1 |
| Phlebitis, | 1 | .... | 1 | .. | .. | .. | .. |
| Paralysis, | 1 | 1 | 2 | .. | 1 | .. | .. |
| Puerperal Convulsions, | .... | 1 | 1 | .. | .. | .. | .. |
| Still-born, | 4 | 2 | 6 | 4 | 2 | .. | .. |
| Suicide, | 1 | 1 | 2 | .. | .. | .. | .. |
| Unknown, | 6 | 5 | 11 | 2 | 2 | .. | .. |
| Ulceration of Bowels, | .... | 1 | 1 | .. | .. | .. | .. |
| Whooping Cough, | 2 | .... | 2 | 1 | .. | .. | .. |
| Totals, | 76 | 54 | 130 | 19 | 15 | 10 | 3 |

NATIVITIES.—American, 47. German, 40. English, 8. Irish, 12. Scotch, 1. Swiss, 1.

*of April,* 1855. By J. Root, M. D., Health Physician.

AGE.

| 2—5 | | 5—10 | | 10—15 | | 15—20 | | 20—25 | | 25—30 | | 30—40 | | 40—50 | | 50—60 | | 60—70 | | 70—80 | | 80—90 | | 90—100 | | 100 and upward. | | Not given. | |
|---|---|---|---|---|---|---|---|---|---|---|---|---|---|---|---|---|---|---|---|---|---|---|---|---|---|---|---|---|---|
| Male. | Female. | Male. | Female. | Male. | Female. | Male. | Female. | Male. | Female. | Male. | Female. | Male. | Female. | Male. | Female. | Male. | Female. | Male. | Female. | Male. | Female. | Male. | Female. | Male. | Female. | Male. | Female. | Male. | Female |
| | | | | | | | | | | | | 1 | | | | | | | | | | | | | | | | | |
| 1 | | | | | | | | | | | | | | | | | | | | | | | | | | | | | |
| | | | | | | | | | | | | 1 | | | | | | | | | | | | | | | | | |
| | | | | | | | | | | | | | | | | | | | | 1 | | | | | | | | | |
| 2 | 1 | | 2 | 3 | | 4 | | | 1 | 1 | | 3 | 1 | | 1 | 1 | 1 | | 1 | | | 1 | | | | | | | |
| 1 | | 1 | 1 | | | | | | | | | | | | | | | | | | | | | | | | | | |
| 1 | 1 | | | | | | | | | | | | | | | | | | | | | | | | | | | | |
| | | | | | | | | | | | | | | | | | | | | | | | | | | | | | |
| | | | | | | | 1 | | | | | | | | | | | | | | | | | | | | | | |
| | | | | | | | | | | | | | | | | | | | | 1 | | | | | | | | | |
| | | | | | | 1 | | | | | | | | | | | | | | | | | | | | | | | |
| 1 | | | | | | | | | | | | | | | | | 1 | | | | | | | | | | | | |
| | 1 | | | | | | | | | | | | | | | | | | | | | | | | | | | | |
| | | | | | | | | | | | | 1 | | 1 | | | | | | | | | | | | | | | |
| | | | | | | | | | | | 1 | | | | | 1 | | | 1 | | | | | | | | | | |
| | | | | | | | | | | | | | | | | | | | | | | | | | | | | | |
| | | 1 | | | | | | | | | 1 | | | | | | 1 | | | | | | | | | | | | |
| | | | | | | | | | | | | | | | | | | | | | | | | | | | | | |
| | | | | | 1 | | | | | | | | | | | | | | | | | | | | | | | | |
| | | | | | | | | | | | | | | | | | | | | | | | | | | | | | |
| | | | | | | | | | | | | | | | | 1 | 1 | | | | | | | | | | | | |
| | | | | | | 1 | | | | | | | | | | | | | | | | | | | | | | | |
| | | | | | | | 1 | | | | | | | | | | | | | | | | | | | | | | |
| 1 | 1 | | | | | | | | | | | | | | | | | | | | | | | | | | | | |
| | | | | | | | | | | | | | 1 | | | | | | | | | | | | | | | | |
| | | | | | | | | | | | | | | 1 | | | | | | | | | | | | | | | |
| | | | | | | | | | | | | | | | | | | | | | | | | | | | | | |
| | | | | | | | | | | | | | 1 | | | | | | | | | | | | | | | | |
| | | | | | | | | | | | | | | | | | | | | | | | | | | | | | |
| | | | | | | | | | | | 1 | | | | | | | | | | | | | | | | | | |
| | | | | | | | | | | | | | | | | | | | | | | | | | | | | | |
| 1 | | | | | | | | | 1 | | | | | | | | | | | | 1 | | | | | | | | |
| | | | | | | | | | | | | 1 | | | | | | | | | | | | | | | | | |
| | | | | | | | | | | | | | | | | | | | 1 | | 2 | | 1 | | | | | | |
| | | 1 | | | | | | | | | | 1 | 1 | 1 | | | | | | | | | | | | | | | |
| | | | | | | | | | | | | 1 | | | | | | | | | | | | | | | | | |
| | | | | | | | | | | | | | | | | 1 | | | | | | | | | | | | | |
| | | | | | | | 1 | | | | | | | | | | | | | | | | | | | | | | |
| | | | | | | | | | | | | | | | | | | | | | | | | | | | | | |
| | 1 | | 1 | | | | | | | 1 | 1 | | | | | | | | | | | | | | | | | | |
| | 1 | | | | | | | | | | | 2 | | 1 | 1 | | 1 | | | | | | | | | | | | |
| | | | | | | | | | | | | | | | | | | | | | | | | | | | | | |
| | | | | | | 1 | | | | | | | | | | | | | | | | | | | | | | | |
| | | | | | | | | | | | | | | | | | | | | | | | | | | | | | |
| | | | | | | | | | | | | | | | | | | | | | | | | | | | | | |
| | | | | | | | | | | | | | | | | | | | | | | | | | | | | | |
| | | | | | | | | | | | | | | | | | | | | | | | | | | | | | |
| | | | | | | | | | | | | | | | | | | | | | | | | | | | | | |
| | | | | | | | | | | | | | | | | | | | | | | | | | | | | | |
| | | | | | | | | | | | | | | | | | | | | | | | | | | | | | |
| | | | | | | | | | | | | | | | | | | | | | | | | | | | | | |
| 8 | 6 | 3 | 4 | 3 | 1 | 7 | 3 | 0 | 2 | 2 | 4 | 11 | 4 | 4 | 2 | 4 | 5 | 0 | 3 | 2 | 3 | 1 | 1 | 0 | 0 | 0 | 0 | 0 | 0 |

French, 5. Canadian, 3. Welsh, 1. Country not given, 12.—Total, 130.

